# An updated systematic review of the association between the TLR4 polymorphism rs4986790 and cancers risk

**DOI:** 10.1097/MD.0000000000031247

**Published:** 2022-10-21

**Authors:** Qiang Xiao, Jian Chen, ShuKun Zeng, Hu Cai, GuoMin Zhu

**Affiliations:** a General Surgery Department, First Affiliated Hospital of Nanchang University, Nanchang, China.

**Keywords:** cancer, SNP, systematic review, TLR4, trial sequential analysis

## Abstract

Toll-like receptor 4 (TLR4) is a lipopolysaccharide receptor that may influence tumor progression through inflammatory response and immune response. This complex process mainly occurs within cells. The correlation between TLR4 and neoplasms has been of great interest, but discrepancies remain.

**Methods:**

We analyze the literature retrieved from five databases (Web of Science, PubMed, Embase, CNKI, and Wan Fang) to assess the intensity of association using odds ratio (ORs) and 95% confidence intervals (95% CI). Meta-regression and subgroup analysis were utilized to find sources of heterogeneity. Publication bias is estimated using contour-enhanced funnel plots, Begg’s test, and Egger’s test, and we implemented sensitivity analysis to clarify the reliability of the outcomes. We also conducted an evaluation of the sample size using trial sequential analysis (TSA) method.

**Results:**

We found a significant association between rs4986790 and tumors (dominant model: OR [95% CI] = 1.25 [1.11–1.42]; heterozygous model OR [95% CI] = 1.25 [1.11–1.41]; and additive model: OR [95% CI] = 1.25 [1.10–1.41]. Specifically, the rs4986790 minor allele G may increase the risk of gastric cancer (dominant model: OR [95% CI] = 1.62 [1.3–2.03]; heterozygous model: OR [95% CI] = 1.57 [1.24–1.97]; additive model: OR [95% CI] = 1.64 [1.31–2.05] and other tumors (dominant model: OR [95% CI] = 1.36 [1.17–1.57]; heterozygous model: OR [95% CI] = 1.43 [1.25–1.63]; additive model: OR [95% CI] = 1.35 [1.18–1.55]. Further subgroup analysis showed that this association are both present in Caucasian and Asian.

**Conclusion:**

The outcomes of our systemic review proved that the TLR4 polymorphism rs4986790 is associated with cancer, especially with gastric cancer, and this strong correlation are evident in Caucasians and Asian.

## 1. Introduction

Cancer has become a common global burden to be alleviated due to its high mortality rate, and cancer has become a severe threat to human health.^[1]^ In 2020 alone, about 20 million new cancer patients and about 10 million cancer deaths are expected worldwide. Although cancer therapy is now more efficient globally, the high incidence and mortality of cancer cannot be ignored.^[2]^ The genetic risk of most cancers is due to multiple risk alleles, each with low to moderate risk.^[3]^ Although rare, mutations in the single nucleotide polymorphisms minor allele are likely to be linked to the development and progression of cancers. Tumor progression involves complicated interplays between tumor cells, immune cells, and the tumor microenvironment.^[4]^ Studies have shown that persistent infection and chronic inflammation can promote many malignancies,^[5]^ such as colorectal, gastric, breast, uterine, liver, and prostate cancers (PCs).^[6–11]^ The potential connection opens up new possibilities for treating cancers associated with chronic persistent infections.

Toll-like receptor 4 (TLRs) are family members of known transmembrane glycoproteins, 13 identified in humans.^[12,13]^ All TLRs are architected as Toll receptors consisting of an external pathogen-associated molecular patterns recognition domain with leucine-rich repeat motifs and an intercellular signaling domain.^[14,15]^ The signaling pathway of TLRs in human individuals originates from the TIR structural domain of the cytoplasm, which is conserved in all TLRs. Due to the resemblance of the cytoplasmic portion of TLRs to the interleukin-1 receptor family, TIR is also known as the Toll/interleukin-1 receptor structural domain. Studies have shown that adapters such as TIRAP, Myd88, TRIF, and TIRP contain TIR domains that modulate the TLR signaling.^[16]^ Myd88 may affect lipopolysaccharide (LPS)-triggered inflammatory responses through adverse regulatory effects.^[17]^ TIRAP binds to TLR4 and participates in the TLR4-mediated Myd88-independent signaling.^[18]^ Compared to MyD88 or TIRAP, overexpression of TRIF triggers IFN-promoter activation and participates in the TLR3-mediated MyD88-independent pathway. TIRP, on the other hand, is a recently discovered adapter containing a TIR domain.^[19]^

Drosophila Toll protein (TLR4) is a representative receptor for Gram-negative LPS of Gram-negative bacteria, which is a critical bridge molecule linking oncogenic infections to cancer.^[5]^ As a vital member of the TLRs family, TLR4 has an extracellular structural domain composed of 22 leucine-rich repeat sequences, which can be expressed in some cells.^[20]^ Upon activation by LPS, LBP translocates to and binds to the TLR4/MD-2 complex via CD14. TIR domain-containing adapters are activated to translocate endotoxin into cells, promoting inflammatory factor production,^[21]^ and exerting its influence on tumorigenesis and development. The polymorphism rs4986790 substitutes the A allele at 896 base pairs by G, resulting in the substitution of glycine for aspartate at amino acid sequence (TLR4_896A/G) site 299. This gene missense mutation alters the extracellular configuration of this receptor and is linked to a blunted response to LPS in vitro and in vivo.^[22]^ Many current studies have suggested a potential relationship between TLR4 polymorphisms and tumors. Zhou et al,^[23]^ Zhao et al,^[24]^ and Zhang et al^[25]^ substantiated that TLR4 may increase gastric cancer (GC) susceptibility, and Li et al^[26]^ revealed that TLR4 plays a crucial role in colorectal carcinogenesis. However, some studies did not suggest an apparent association.^[27–29]^ Because of these conflicting conclusions, we are undertaking the most extensive systematic review, enhancing study precision with large sample sizes.

## 2. Materials and Methods

This meta-analysis was prospectively registered on PROSPERO. We developed the study protocol according to the PRISMA guidelines. This study does not directly involve animal or human trials and does not require an ethics approval statement.

### 2.1. Search strategy and eligibility criteria

#### 2.1.1. Search and selection of included studies.

Two authors searched the literature from five web databases (Web of Science, PubMed, Embase, CNKI, and Wan Fang database), independently retrieved and selected appropriate records, and used Endnote software to categorize and organize related literature. The Searched pieces of literature are published until March 2022, without language or geographical restrictions. The search formula will comprise the below terms: “Toll-Like Receptor 4,” “TLR4 receptor,” “rs4986790,” “Neoplasia,” “Tumor,” “Cancer,” “Malignancy,” “Carcinoma,” “SNPs,” “Polymorphism,” and “Single Nucleotide Polymorphisms”(See File S1, http://links.lww.com/MD/H680, Supplemental Digital Content, which demonstrates specific search strategies). We also ensured that some studies that might have been missed were selected by manually searching the reference citations included in the article. If the criteria are met, some of the gray studies will be included as study data, Including dissertation, preprint, Etc. If there is any inconsistency between two authors during the search process, a third author must be involved until the problem is resolved.

#### 2.1.2. Eligibility criteria.

Separate searches of the included literature by both authors were required to comply with the following conditions: were a case-control study; evaluated the correlation between TLR4 polymorphism (“4986790”) and cancers risk; each tumor diagnosis meets the diagnostic criteria; had available genotype frequencies both in cases and controls. Articles that do not meet the appeal criteria will be excluded. Of the multiple studies for which duplicate data were published, only the most current or intact study was selected.

### 2.2. Data extraction

Two investigators (QX and JC) involved in this study respectively extracted the following required contents from each of the 39 initially included papers (12,694 vs, 16,371 controls): first author, publication year, country region, ethnicity, source of the control group, cancer type, and genotype frequency of case and control groups, genotyping method. The Hardy-Weinberg equilibrium (HWE) was calculated with data from each study control group. *P*-values based on the χ^2^ test will be applied to weigh the HWE results, and studies with *P* > .05 will be consistent with HWE. Studies that do not comply with HWE will be excluded.

### 2.3. Literature quality evaluation

The quality results of the incorporated articles will undoubtedly affect the conclusions of our study. Therefore, we applied the Newcastle-Ottawa Scale (NOS) to estimate study quality. Two authors evaluated the incorporated studies separately, integrated the final results, and settled them by negotiation when inconsistencies arose; if essential, consider involving a third party until the issue was resolved. The evaluation form, with a total score of 9, is developed in three aspects: selection, comparability, and exposure. A total NOS score greater than six will be considered a high-quality study. Otherwise, it is of low quality (see Table S1, Supplemental Digital Content, http://links.lww.com/MD/H681, Supplemental Digital Content, which aggregates the quality of all included literature).

### 2.4. Statistical analysis

We used STATA 15 as the data processing software for the research process. In five models, investigators used pooled odds ratio (ORs) and 95% confidence intervals (CIs) to evaluate the connection between TLR4 polymorphism (rs4986790) and cancer risk

#### 2.4.1. Heterogeneity analysis and subgroup analysis.

We used a chi-square-based *Q*-test and *I*-square to assess the heterogeneity level. *P*-values less than .1 or *I*-square greater than 50% were identified as high heterogeneity. For studies with high heterogeneity, we will apply a random-effect model to estimate the association between rs4986790 and cancers; otherwise, a fixed-effect model will be adopted. If heterogeneity exists, we will conduct meta-regression to find the origin of heterogeneity and further explore the specific origin of heterogeneity through subgroup analysis.

#### 2.4.2. Sensitivity analysis and publication bias.

We apply sensitivity analysis to judge the reliability of the stability of the results. We eliminated sequentially one case-control study and analyzed the rest of the studies to compare whether the result varied from the initial result. If the results changed, then this study may have been excluded. This approach will make our study more credible. We evaluated the risk of publication bias with contour-enhanced funnel plots, Begg’s, and Egger’s tests. Publication bias was present if the white area of the funnel plot was asymmetric or if the *P*-value of the Begg’s and Egger’s tests were less than 0.05.

### 2.5. False-positive report probability analysis and trial sequential analysis

We evaluated the results using false-positive reporting probability (FPRP) analysis, where the FPRP value is determined by the observed *P*-value, statistical efficacy, and applicable prior probability. We set the FPRP threshold to 0.2 and examine the dominance OR of 0.67/1.50 (protection/risk effect) with a priori probability of 0.1. Results will be significant if the FPRP value is less than 0.2.^[30]^ The trial sequential analysis (TSA) results represent the sample size required for the current conclusions drawn from the meta-analysis. They can rectify the enhanced risk of type I and type II errors from multiple updates of the meta-analysis, which are mainly due to data merging.^[31]^ The application of TSA minimizes false-positive results resulting from random errors when the sample size of meta-analysis does not reach a sufficient sample size. The TSA software (0.9.5.10 Beta) is what we used. We run the software in strict accordance with the TSA user manual,^[32]^ which is available at www.ctu.dk/tsa. We used a type I error of 5%, a type II error of 20% (power of 80%), and the traditional cutoff value (*Z* value) of 1.96 (α = 0.05). The results were described synthetically by the relationship between the cumulative *Z*-value and the TSA boundary curve. If the cumulative *Z*-value reached the information size or transcended the TSA boundary curve, the sample size met the required number for the study; otherwise, the study required a more extensive original study to substantiate the results further.^[33,34]^

### 2.6. In-silico analysis of TLR4

We applied an open interactive website called GEPIA (http://gepia.cancer-pku.cn/) to analyze the differences in RNA sequencing expression data between tumor and normal tissues.^[34–36]^ GEPIA data are derived from the TCGA and GTEx databases and can further explore the association between TLR4 and cancer.^[37]^ We present some of the results in the form of boxplots.

## 3. Result

### 3.1. Screening process

**Figure 1.**
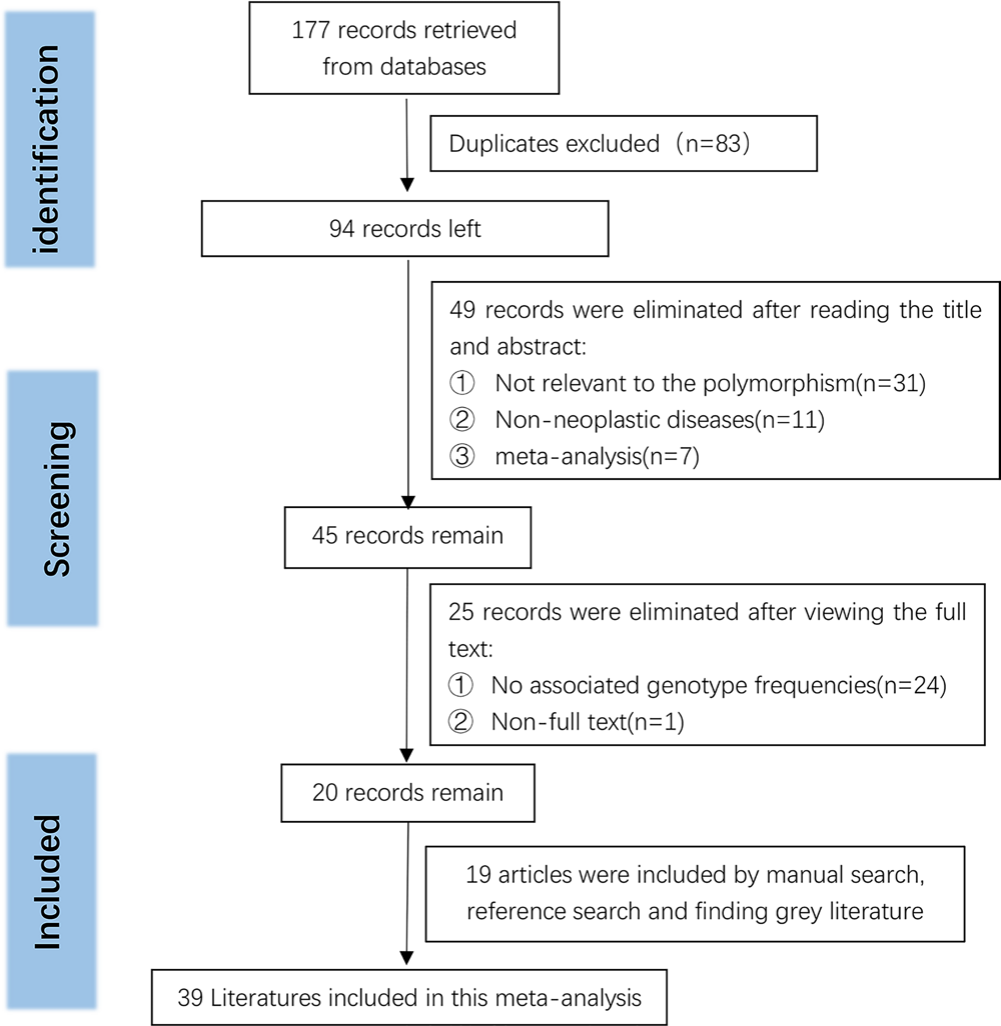
Flow diagram of literature screening.

We retrieved 177 records that matched the conditions according to the method mentioned above (PubMed n = 23, WOS n = 47, Embase n = 65, CNKI n = 3, wan fang n = 42). We eliminated 83 duplicate records by literature management software (Endnote X9). Subsequently, we read the titles and abstracts of the literature and realized that there were still 49 articles that did not conform to the requirements. Full-text browsing of the remaining literature was continued, removing some of the literature, most of which had no acquired genotype frequencies. We also manually searched for reference citations in incorporated articles and similar articles. A summary of 19 articles was additionally selected. Researchers excluded three ineligible studies after assessing HWE based on control group data. The final number of included studies are 38 (Fig. 1).

### 3.2. Features of the included studies

We extracted relevant content from 38 studies including 12,694 cases and 16,371 controls. The year of publication of the included literature is from 2004 to the present, and basically, all relevant studies are included. One of all incorporated papers was a dissertation. A wide range of tumors are involved, which encompass PC,^[38–44]^ GC,^[27,45–53]^ colorectal cancer,^[28,29,54–57]^ non-Hodgkin’s lymphoma,^[58–60]^ breast cancer,^[61]^ lung cancer,^[62]^ and other tumors.^[46,63–73]^ The ethnic distribution of subjects is mainly Caucasian(n = 31) and Asian(n = 5). Some studies did not make a precise distinction between the ethnic groups, and we defined these as mixed ethnic groups (n = 2). The control population was obtained from the community and hospital by a randomized method. For the accuracy of the experiment, we have excluded studies that did not comply with HWE. In contrast, all included literature had quality assessment (NOS) scores above five, and all studies could be considered high quality (Table 1).

**Table 1:** Literature extraction information in tabular form.

Author	Year	Country	Ethnicity	Control source	Cancer type	AA/AG/GG		Genotyping method	HWE	NOS
						Case	Control			
Zheng^[38]^	2004	Sweden	Caucasian	PB	PC	1241/136/1	693/79/5	MassARRAY	0.10	7
Hellmig^[63]^	2005	Germany	Caucasian	PB	CML	83/4/0	837/114/1	Taqman	0.15	6
Chen^[39]^	2005	US	Caucasian	PB	PC	588/66/3	605/59/5	Sequenom	0.01	8
Boraska^[54]^	2006	Croatia	Caucasian	PB	CRC	77/10/2	84/4/0	PCR-RFLP	0.83	7
Landi^[29]^	2006	Italy	Caucasian	HB	CRC	251/31/0	232/37/0	TaqMan	0.23	6
Forrest^[58]^	2006	US/UK	Caucasian	PB	NHL	794/106/3	1254/172/6	TaqMan	0.97	7
Nieters^[59]^	2006	German	Caucasian	PB	HNL	258/39/1	596/71/1	PCR-RFLP	0.46	7
Garza-Gonzalez^[45]^	2007	Mexico	Mixed	NA	GC	72/6/0	175/14/0	PCR-RFLP	0.60	6
Hold^[46]^ (A-group)	2007	Polish	Caucasian	PB	GC	258/51/3	387/31/1	Taqman	0.65	7
(B-group)	2007	US	Caucasian	PB	GC	266/38/3	194/16/1	Taqman	0.30	7
(C-group)	2007	US	Caucasian	PB	ESCA	97/10/0	194/16/1	Taqman	0.30	7
Cheng^[40]^	2007	US	Mixed	HB	PC	439/66/1	456/48/2	Taqman	0.54	6
Santini^[27]^	2008	Rome	Caucasian	PB	GC	159/11/1	140/11/0	allele- PCR	0.64	7
Ture-Ozdemir^[64]^	2008	Greece	Caucasian	HB	CML	38/18/0	39/12/0	PCR-RFLP	0.34	6
Trejo-de la^[47]^	2008	Mexico	Mixed	HB	GC	34/4/0	138/6/0	allele- PCR	0.80	6
Wang^[41]^	2009	US	Caucasian	PB	PC	230/24/0	216/35/0	Taqman	0.24	7
Etokebe^[61]^	2009	Croatia	Caucasian	HB	BC	110/20/0	84/15/0	TaqMan	0.41	6
Pandey^[65]^	2009	India	Asian	NA	CC	114/35/1	123/26/1	PCR-RFLP	0.78	6
Purdue^[60]^	2009	US	Caucasian	PB	NHL	1195/133/6	1126/131/8	Illumina	0.06	7
Balistreri^[42]^	2010	Italy	Caucasian	PB	PC	49/1/0	111/13/1	PCR-RFLP	0.38	7
Ashton^[66]^	2010	Australia	Caucasian	PB	EC	163/25/3	258/31/2	Sequenom	0.33	7
Gast^[67]^	2011	German	Caucasian	PB	MM	665/91/0	659/73/3	Sequenom	0.53	6
Davoodi^[55]^	2011	Malaysia	Asian	HB	CRC	58/2/0	49/1/0	PCR-RFLP	0.94	6
Shui^[43]^	2012	US	Caucasian	PB	PC	1152/131/3	1126/136/5	Sequenom	0.68	7
de Oliveira^[48]^	2012	Brazil	Caucasian	HB	GC	154/20/0	215/10/0	PCR-RFLP	0.73	6
de Oliveira^[49]^	2013	Brazil	Caucasian	HB	GC	174/26/0	224/16/0	PCR-RFLP	0.59	6
Qadri^[50]^	2013	India	Asian	PB	GC	107/23/0	169/31/0	PCR-RFLP	0.23	6
Shen^[68]^	2013	China	Asian	HB	BLC	431/2/3	519/1/2	PCR-RFLP	<0.01	6
Pimentel-Nunes^[56]^	2013	Portugal	Caucasian	HB	CRC	169/15/0	186/5/0	PCR-RFLP	0.85	6
Kutikhin^[52]^	2014	Russia	Caucasian	PB	GC	46/11/0	258/39/0	Taqman	0.23	8
Companioni^[51]^	2014	Spain	Caucasian	PB	GC	316/45/0	1134/133/3	Illumina	0.66	7
Omrane^[57]^	2014	Tunis	Caucasian	HB	CRC	87/13/0	120/18/2	SNaPshot	0.18	6
Kurt^[62]^	2016	Turkey	Caucasian	HB	LC	159/1/0	99/1/0	Sequenom	0.96	6
Li^[44]^	2017	China	Asian	HB	PC	78/14/4	71/13/3	PCR-RFLP	0.03	6
Pandey^[69]^	2019	India	Asian	HB	CC	70/37/3	107/32/2	PCR-RFLP	0.82	6
Aref^[70]^	2020	Egypt	Caucasian	NA	AML	84/30/6	86/12/2	PCR-RFLP	0.06	7
Eed^[53]^	2020	Saudi Arabia	Caucasian	NA	GC	26/10/9	56/21/3	PCR-RFLP	0.57	6
Neamatallah^[71]^	2020	Egypt	Caucasian	PB	HCC	221/95/17	744/263/21	TaqMan	0.69	7
Quirino^[72]^	2021	Brazil	Caucasian	PB	MPN	148/19/0	202/20/0	PCR-RFLPA	0.48	6
Reilly^[28]^	2021	Ireland	Caucasian	HB	CRC	25/5/0	45/10/0	Amplifluor	0.46	6
Banescu^[73]^	2022	Romania	Caucasian	PB	AML	470/40/1	473/30/0	TaqMan	0.49	7

Red font in Table 1 indicates that the *P* value of HWE is less than .05.

AML = acute myeloid leukemia, BC = breast cancer, BLC = bladder cancer, CC = cervical cancer, CML = gastric mucosa‑associated lymphoid tissue (MALT) lymphoma, CRC = colorectal cancer, EC = endometrial cancer, ESCA = esophageal carcinoma, GC = gastric cancer, HB = hospital-based, HCC = hepatocellular carcinoma, HWE = Hardy–Weinberg equilibrium, LC = lung cancer, **Mixed:** subjects were from two or more races, MM = malignant melanoma, MPN = myeloproliferative neoplasms, NA = control group source unknown, NHL = non-Hodgkin lymphoma, PB = population-based, PC = prostate cancer.

### 3.3. RS4986790 (A > G)

**Figure 2.**
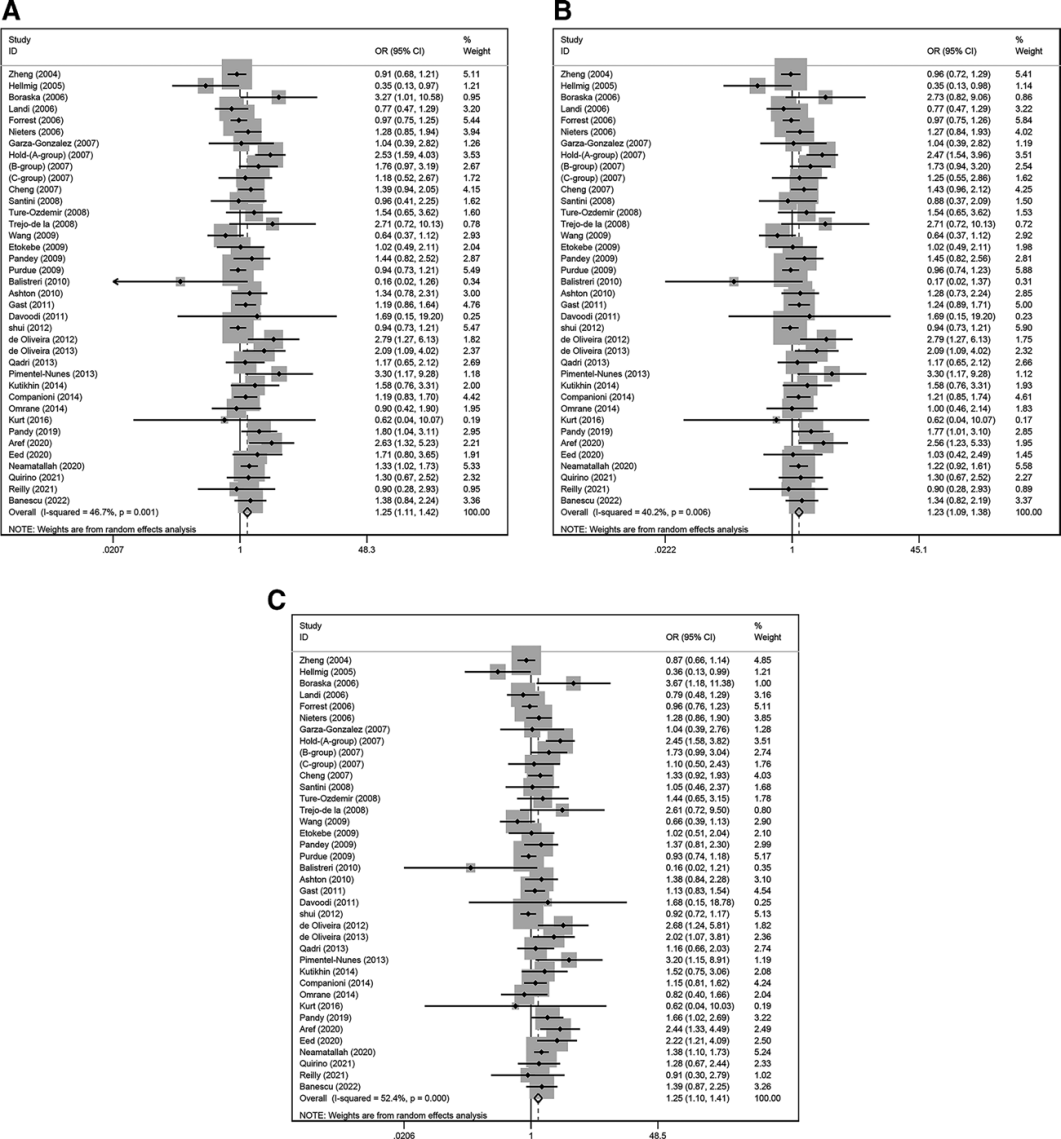
Forest plots of the three models regarding the correlation between TLR4 and cancer susceptibility. (A) Correlation of rs4986790 with cancers susceptibility in dominant model. (B) Correlation of rs4986790 with cancers susceptibility in heterogeneous model. (C) Correlation of rs4986790 with cancers susceptibility in additive model.

Our data analysis indicates that the G allele of rs4986790 is associated with the development of cancers (Fig. 2, dominant model: OR [95% CI] = 1.25 [1.11–1.42]; heterozygous model: OR [95% CI] = 1.25 [1.11–1.41]; additive model: OR [95% CI] = 1.25 [1.10–1.41]). Of all the models analyzed, the recessive and homozygous use a fixed-effects model, while the rest use a random-effects model. We then looked for heterogeneity by meta-regression and discovered that the heterogeneity was mainly derived from genotyping method subgroup. Further subgroup analysis suggested that the heterogeneity originated from studies in which the genotyping method was Taqman (dominant model: *P*_h_ < .01, *I*^2^ = 63.0%; heterozygous model: *P*_h_ < .01, *I*^2^ = 60.1%; additive model: *P*_h_ < .01, *I*^2^ = 64.1%), while no distinct heterogeneity was found in other subgroups (Table 2).

**Table 2:** Outcomes of data analysis on the relationship between TLR4 and cancers.

SNPs	Dominant model		Recessive model		Homozygous model		Heterozygous model		Additive model	
	OR (95% CI)	*P*/*I*^2^ (%)	OR (95% CI)	*P*/*I*^2^ (%)	OR (95% CI)	*P*/*I*^2^ (%)	OR (95% CI)	*P*/*I*^2^ (%)	OR (95% CI)	*P*/*I*^2^ (%)
rs4986790 (A > G)	GG + AG vs AA		GG vs AG + AA		GG vs AA		AG vs AA		G vs A	
Overall	**1.25 (1.11, 1.42**)	**<.01/46.7%**	1.16 (0.79, 1.7)	.414/3.4%	1.19 (0.81, 1.75)	.37/6.7%	**1.25 (1.11, 1.41**)	**<.01/45.1%**	**1.25 (1.10, 1.41**)	**<.01/52.4%**
Model	R		F		F		R		R	
Subgroup										
Ethnicity										
Caucasian	**1.22 (1.06, 1.41**)	**<.01/53.0%**	1.16 (0.77, 1.74)	.282/14.0%	1.18 (0.79, 1.77)	.252/16.5%	**1.22 (1.06, 1.4**)	**<.01/52.2%**	**1.23 (1.07, 1.42**)	**<.01/58.9%**
Asian	**1.47 (1.06, 2.03**)	**.776/0.0%**	1.60 (0.36, 7.2)	.693/0.0%	1.83 (0.4, 8.26)	.657/0.0%	**1.46 (1.05, 2.02**)	**.803/0.0%**	**1.41 (1.05, 1.9**)	**.813/0.0%**
Mixed	1.41 (0.99, 2.0)	.522/0.0%	0.5 (0.05, 5.52)	–/–	0.52 (005, 5.75)	–/–	**1.44 (1.01, 2.05**)	**.525/0.0%**	1.35 (0.97, 1.89)	.526/0.0%
Control source										
PB	1.15 (1.0, 1.32)	<.01/51.1%	0.89 (0.55, 1.42)	.639/0.0%	1.50 (1.07, 2.08)	.216/20.4%	**1.16 (1.01, 1.34**)	**<.01/53.8%**	1.14 (0.99, 1.32)	<.01/56.9%
HB	**1.44 (1.11, 1.87**)	**.148/29.5%**	0.85 (0.25, 2.84)	.647/0.0%	0.85 (0.25, 2.84)	.467/0.0%	**1.45 (1.13, 1.87**)	**.178/26.3%**	**1.38 (1.08, 1.77**)	**.149/29.5%**
NA	**1.68 (1.18, 2.39**)	**.419/0.0%**	**3.64 (1.39, 9.51**)	**.435/0.0%**	**3.64 (1.39, 9.51**)	**.435/0.0%**	**1.50 (1.02, 2.23**)	**.351/8.4%**	**1.76 (1.24, 2.51**)	**.296/18.9%**
Cancer type										
PC	0.93 (0.71, 1.22)	.073/53.3%	0.38 (0.14, 1.01)	.599/0.0%	0.38 (0.14, 1.0)	.606/0.0%	0.95 (0.72, 1.25)	.073/53.2%	0.91 (0.71, 1.18)	.082/51.7%
GC	**1.62 (1.3, 2.03**)	**.231/22.3%**	**3.29 (1.38, 7.81**)	**.613/0.0%**	**3.36 (1.4, 8.03**)	**.623/0.0%**	**1.57 (1.24, 1.97**)	**.213/24.2%**	**1.64 (1.31, 2.05**)	**.165/29.5%**
CRC	1.32 (0.75, 2.33)	.074/50.2%	1.19 (0.23, 6.21)	.186/42.8%	1.23 (0.24, 6.38)	.175/45.6%	1.29 (0.76, 2.19)	.122/42.5%	1.33 (0.74, 2.38)	.046/55.8%
NHL	1.0 (0.79, 1.26)	.152/40.4%	0.86 (0.39, 1.88)	.712/0.0%	0.86 (0.39, 1.88)	.724/0.0%	1.0 (0.79, 1.27)	.172/37.3%	0.99 (0.79, 1.24)	.159/39.4%
Others	**1.36 (1.17, 1.57**)	**.784/0.0%**	1.4 (0.65, 2.97)	.704/0.0%	1.53 (0.72, 3.25)	.664/0.0%	**1.43 (1.25, 1.63**)	**.853/0.0%**	**1.35 (1.18, 1.55**)	**.736/0.0%**
Genotyping methods										
PCR-RFLP	**1.63 (1.33, 2.0**)	**.296/1.4%**	**2.99 (1.42, 6.28**)	**.831/0.0%**	**3.21 (1.51, 6.75**)	**.851/0.0%**	**1.57 (1.28, 1.92**)	**.331/10.9%**	**1.64 (1.33, 2.01**)	**.199/23.0%**
Sequenom	1.06 (0.88, 1.27)	.534/0.0%	0.70 (0.26, 1.83)	.235/30.9%	0.7 (0.27, 1.85)	.23/32%	1.06 (0.88, 1.28)	.523/0.0%	1.03 (0.86, 1.23)	.456/0.0%
Taqman	1.18 (0.93, 1.49)	<.01/63.0%	1.31 (0.59, 2.91)	.802/0.0%	1.34 (0.61, 2.98)	.787/0.0%	1.17 (0.93, 1.46)	<.01/60.1%	1.18 (0.94, 1.47)	<.01/64.1%
Allele-PCR	1.42 (0.53, 3.8)	.195/40.5%	2.67 (0.11, 65.93)	–/–	2.64 (0.11, 65.39)	–/–	1.38 (0.47, 4.05)	.162/48.8%	1.43 (0.61, 3.38)	.239/27.8%
MassARRAY	0.91 (0.68, 1.21)	–/–	0.11 (0.01, 0.96)	–/–	0.11 (0.01, 0.96)	–/–	0.96 (0.72, 1.29)	–/–	0.87 (0.66, 1.14)	–/–
Illumina	1.02 (0.82, 1.26)	.3/7.0%	0.68 (0.25, 1.83)	.828/0%	0.68 (0.25, 1.83)	.841/0.0%	1.04 (0.83, 1.3)	.289/10.9%	1.0 (0.82, 1.21)	.329/0.0%
SNaPshot	0.9 (0.42, 1.9)	–/–	0.28 (0.01, 5.8)	–/–	0.28 (0.01, 5.81)	–/–	1.0 (0.46, 2.14)	–/–	0.82 (0.41, 1.66)	–/–
Amplifluor	0.9 (0.28, 2.93)	–/–	–/–	–/–	–/–	–/–	0.9 (0.28, 2.93)	–/–	0.91 (0.30, 2.79)	–/–

The bold values represent significant results.

CI = confidence interval, CRC = colorectal cancer, GC = gastric Cancer, HB = hospital-based, NA = control group source unknown, NHL = non-Hodgkin lymphoma, OR = odds ratio, PB = population-based, PC = prostate cancer, SNPs = single nucleotide polymorphisms, TLR4 = toll-like receptor 4.

In a subgroup analysis of ethnicity, we can conclude that a strong association between rs4986790 and tumors was seen in both Caucasian (dominant model: OR [95% CI] = 1.22 [1.06–1.41]; heterozygous model: OR [95% CI] = 1.22 [1.06–1.40]; additive model: OR [95% CI] = 1.23 [1.07–1.42] and Asian (dominant model: OR [95% CI] = 1.47 [1.06–2.03]; heterozygous model: OR [95% CI] = 1.46 [1.05–2.02]; additive model: OR [95% CI] = 1.41 [1.05–1.9]), In contrast, this association is not distinctive in the mixed group population. And in the subgroup analysis of tumors, we found that rs4986790 is significantly associated with the risk of GC (dominant model: OR [95% CI] = 1.62 [1.3–2.03]; heterozygous model: OR [95% CI] = 1.57 [1.24–1.97]; additive model: OR [95% CI] = 1.64 [1.31–2.05]) and other cancers (dominant model: OR [95% CI] = 1.36 [1.17–1.57]; heterozygous model: OR [95% CI] = 1.43 [1.25–1.63]; additive model: OR [95% CI] = 1.35 [1.18–1.55]). For subgroup analyses of control group sources, we can conclude that population-based (dominant model: OR [95% CI] = 1.15 [1.0–1.32]; heterozygous model: OR [95% CI] = 1.16 [1.01–1.34]), hospital-based (dominant model: OR [95% CI] = 1.44 [1.11–1.87]; heterozygous model: OR [95% CI] = 1.45 [1.13–1.87]; additive model: OR [95% CI] = 1.38 [1.08–1.77]), and unknown populations (dominant model: OR [95% CI] = 1.68 [1.18–2.39]; heterozygous model: OR [95% CI] = 1.50 [1.02–2.23]; additive model: OR [95% CI] = 1.76 [1.24–2.51]) all manifested the connection between the TLR4 polymorphism rs4986790 and cancers.

### 3.4. Sensitivity analysis and bias

**Figure 3.**
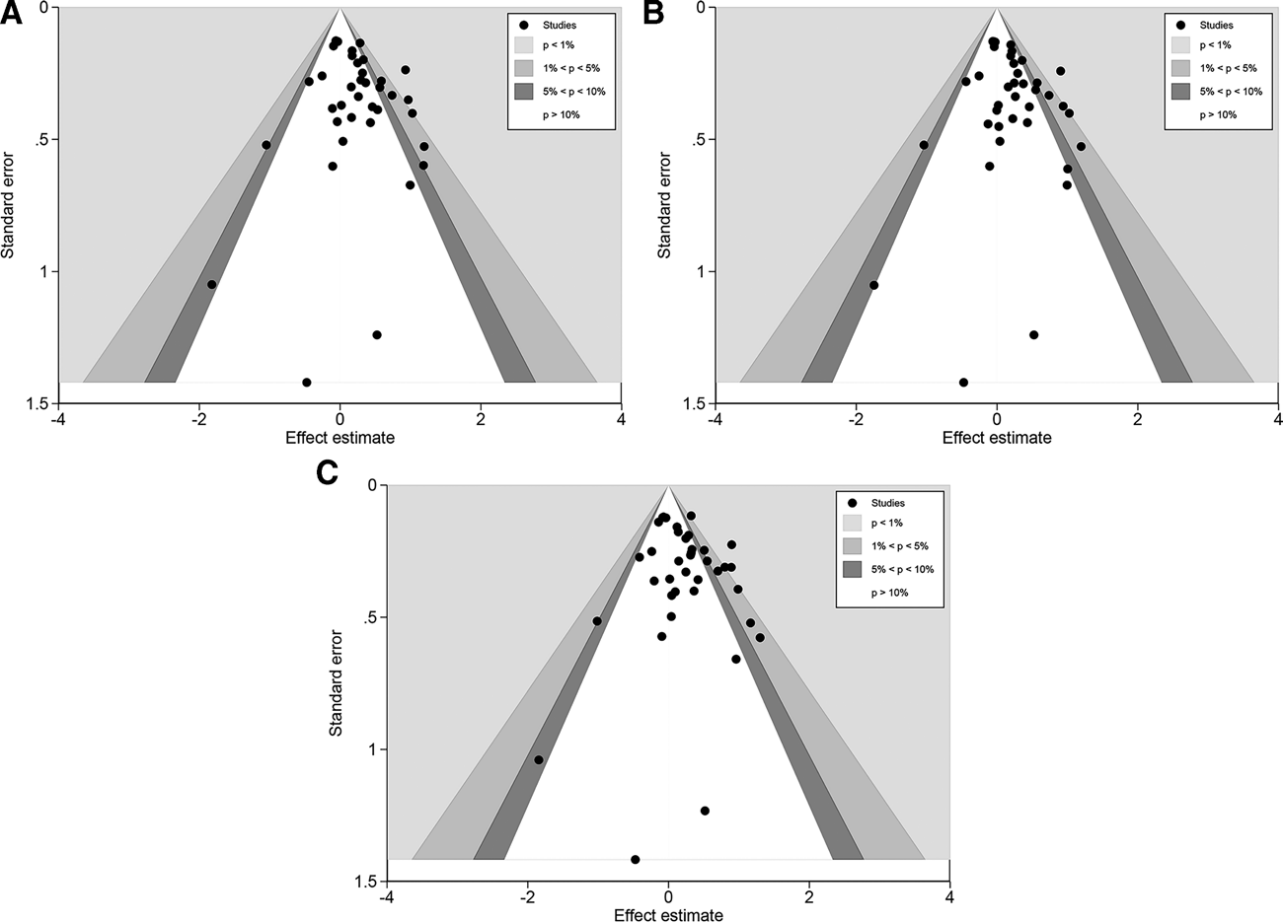
Contour-enhanced funnel plots of the three models. (A) Dominant model; (B): heterogeneous model; and (C) additive model.

**Figure 4.**
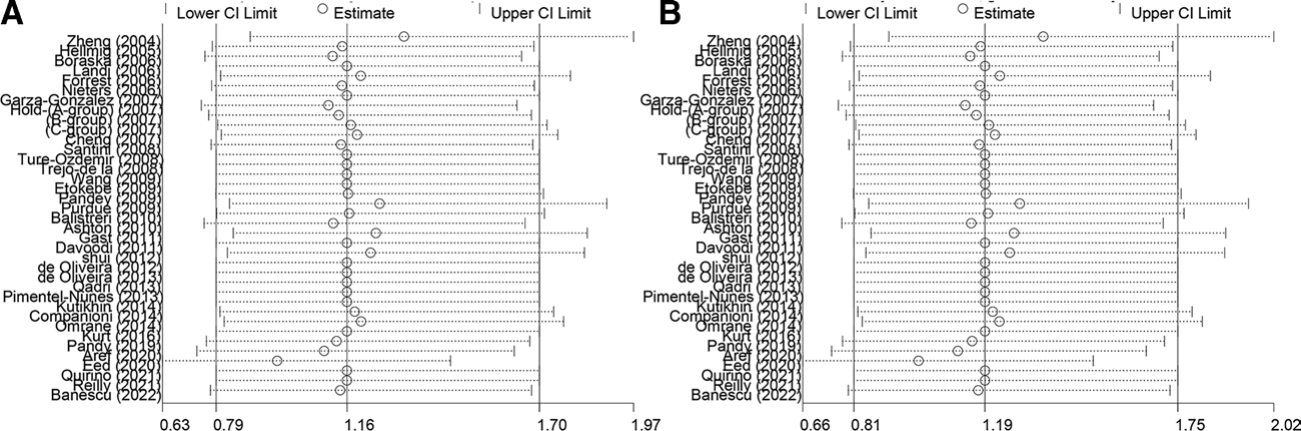
More robust results for both models after removal of one study. (A) Recessive model and (B) homozygous model.

We assessed the publication bias of the literature by employing contour-enhanced funnel plots and the presence of significant asymmetry suggested bias. The final contour-enhanced funnel plot did not suggest significant dissymmetry (Fig. 3). To compensate for the error caused by subjective factors, we further performed quantitative tests for publication bias to verify whether publication bias exists. The result is that no publication bias was shown by Begg’s or Egger’s test (see Table S2, Supplemental Digital Content, http://links.lww.com/MD/H682, Supplemental Digital Content, which demonstrates the results of quantitative analysis of publication bias in the included literature). Conducting sensitivity analyses can estimate the stability and credibility of study results. In the study, we found that the results (pooled ORs) changed after removing one study by Neamatallah et al in the recessive and homozygous models, so we excluded this study (Fig. 4).

### 3.5. Result of FPRP and TSA

**Figure 5.**
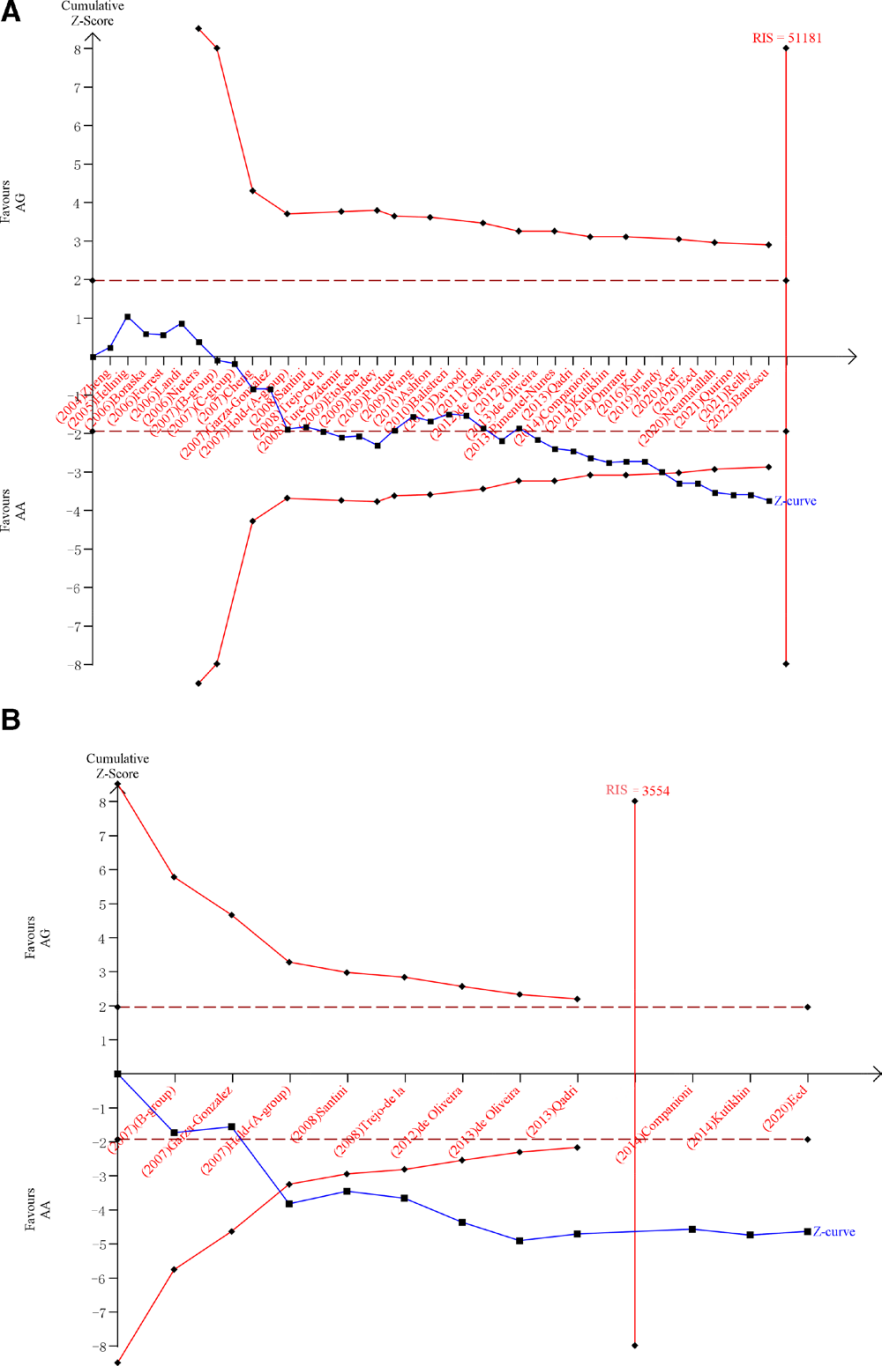
(A) Sample size assessment based on heterogeneous model for all tumor types. (B) Sample size assessment based on heterogeneous model for gastric cancer.

Table 3 reveals the FPRP values calculated at six a priori probability levels. All FPRP values are less than 0.2 at the prior probability level of 0.1, both overall and in subgroups, implying that this finding is noteworthy. We can derive TSA information from Figure 5. Our TSA is based on the heterogeneous model. In the sample size assessment for all tumor types (Fig. 5A), the required information size is 51,181, the absolute value of the cumulative *Z*-value exceeds 1.96 (α > 0.05), and *Z*-curve intersects with the TSA boundary curve. However, it is worth mentioning that the number of cases corresponding to the cumulative *Z* value did not exceed the required information. In the sample size assessment of GC (Fig. 5B), the required information size was 3554, the absolute value of the cumulative *Z* value exceeded 1.96 (α > 0.05), and *Z*-curve intersects with the TSA boundary curve. In addition, the number of cases corresponding to the cumulative *Z* value exceeded the required information size.

**Table 3:** FPRP values calculated at six prior probability levels.

Model	Subgroup	*P* value	OR (95% CI)	Statistical power	Prior probability					
					0.25	0.1	0.01	0.001	0.0001	0.00001
GG + AG vs AA	Overall	<.001	1.25 (1.11, 1.42)	0.997	0.002	0.005	0.057	0.377	0.858	0.984
	Caucasian	.007	1.22 (1.06, 1.41)	0.997	0.021	0.060	0.413	0.877	0.986	0.999
	Asian	.019	1.47 (1.06, 2.03)	0.549	0.095	0.241	0.777	0.972	0.997	1.000
	GC	<.001	1.62 (1.3, 2.03)	0.252	<0.001	0.001	0.011	0.099	0.524	0.917
	Others	<.001	1.36 (1.17, 1.57)	0.909	<0.001	<0.001	0.003	0.029	0.229	0.748
	HB	.006	1.44 (1.11, 1.87)	0.620	0.029	0.083	0.499	0.909	0.990	0.999
AG vs AA	Overall	<.001	1.25 (1.11, 1.41)	0.998	0.001	0.003	0.027	0.220	0.739	0.966
	Caucasian	.005	1.22 (1.06, 1.40)	0.998	0.014	0.040	0.314	0.822	0.979	0.998
	Asian	.022	1.46 (1.05, 2.02)	0.565	0.106	0.262	0.797	0.975	0.997	1.000
	GC	<.001	1.57 (1.24, 1.97)	0.347	0.001	0.003	0.027	0.220	0.739	0.966
	Others	<.001	1.43 (1.25, 1.63)	0.763	<0.001	<0.001	<0.001	<0.001	0.001	0.011
	HB	.004	1.45 (1.13, 1.87)	0.603	0.020	0.059	0.408	0.874	0.986	0.999
G vs A	Overall	<.001	1.25 (1.10, 1.41)	0.998	0.001	0.003	0.027	0.220	0.739	0.966
	Caucasian	.005	1.23 (1.07, 1.42)	0.997	0.014	0.041	0.320	0.826	0.979	0.998
	Asian	.024	1.41 (1.05, 1.9)	0.658	0.098	0.247	0.783	0.973	0.997	1.000
	GC	<.001	1.64 (1.31, 2.05)	0.217	<0.001	0.001	0.006	0.060	0.391	0.865
	Others	<.001	1.35 (1.18, 1.55)	0.933	<0.001	<0.001	0.002	0.022	0.181	0.689
	HB	.011	1.38 (1.08, 1.77)	0.744	0.043	0.119	0.598	0.938	0.993	0.999

The recessive and homozygous model were not statistically significant and therefore were not included in the calculation of the of false-positive report probability.

CI = confidence interval, FPRP = false-positive reporting probability, GC = gastric cancer, HB = hospital based, OR = odds ratio

### 3.6. In-silico analysis

**Figure 6.**
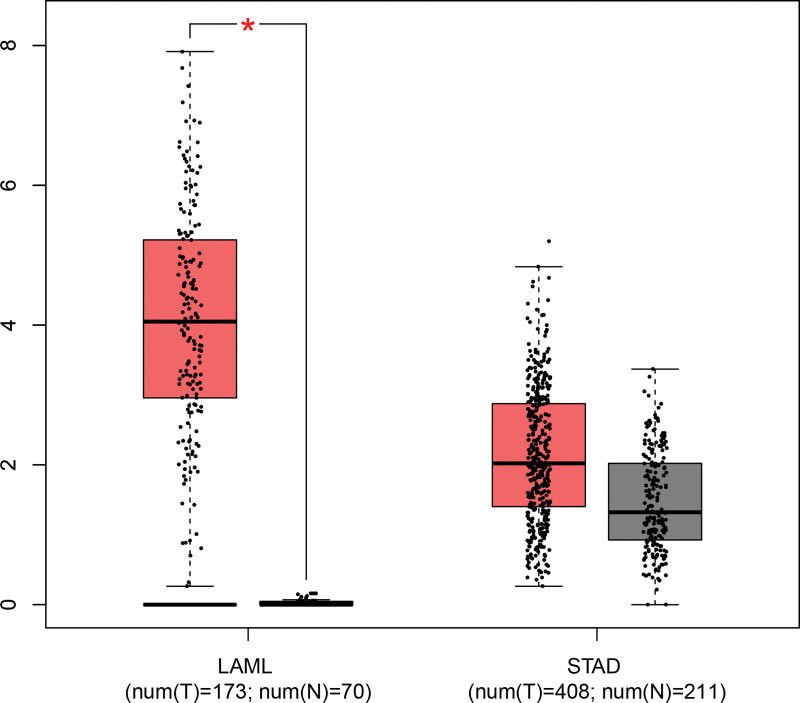
Results of in silico analysis of TLR4 at high expression in different cancer tissues. LAML = acute myeloid leukemia, STAD = stomach adenocarcinoma, TLR4 = toll-like receptor 4.

The results of in-silico analysis suggested that the expression of TLR4 in tumor tissues was significantly higher in acute myeloid leukemia than in normal tissues. Although the expression of TLR4 was more elevated in stomach adenocarcinoma tissues than in normal tissues, this discrepancy was not significant (Fig. 6).

## 4. Discussion

Our meta-analysis discussed the connection between TLR4 polymorphism (rs4986790) and tumors. We showed that rs4986790 increased the risk of GC, while no significant association was seen for colorectal cancer, PC, and non-Hodgkin’s lymphoma. This result is congruent with the conclusion of Zhou et al,^[23]^ Zhao et al,^[24]^ and Zhang et al.^[25]^

TLR4, as an LPS receptor, may promote inflammatory and immune processes through antigen-presenting cell responses.^[74]^ It is worth affirming that the inflammatory response is an influential factor in cancer progression. Previously, TLR4 polymorphisms have been extensively studied, with rs4986790 being the most studied. Several studies have suggested an association between TLR4 and cancers, but some studies have not suggested the same results. Because of the inconsistency of these studies, we undertook this updated systematic review. This systematic review included 38 studies (29,065 subjects), the largest sample size to date, and the results are likely to be more credible. In addition, there were 13 tumor types included in our meta-analysis. Five studies examined the association of TLR4 polymorphism rs4986790 with PC, and six studies for non-Hodgkin’s lymphoma. There were 6 and 11 studies involving colorectal and GCs, separately. Others include acute myeloid leukemia,^[73]^ bladder cancer,^[68]^ breast cancer,^[61]^ cervical cancer,^[65]^ endometrial cancer,^[66]^ malignant melanoma,^[67]^ lung cancer,^[62]^ liver cancer,^[71]^ and chronic myeloproliferative neoplasm^[72]^ also included in our meta-analysis. However, each of them has less than two research articles. Explore as much as possible the relationship between tumors and TLR4.

According to the previous results, the heterogeneity detected by meta-regression analysis mainly stemmed from the genotyping method (see Table S3, Supplemental Digital Content, http://links.lww.com/MD/H683, Supplemental Digital Content, which shows a preliminary appraisal of sources of heterogeneity using meta-regression). Further subgroup analysis suggested significant heterogeneity in the Taqman subgroup, which may be due to potential genotyping method errors, the undeniable effects of chance errors, and experimental design methods; this may not be completely avoidable. The asymmetry in the white areas was found when assessing publication bias based on funnel plots. While neither Begg’s test nor Egger’s test showed publication bias, it led us to consider that this asymmetry was due to heterogeneity. In the meantime, we removed language restrictions during the literature record screening to minimize selection bias. For sensitivity analysis, we found an individual study in the Homozygous and recessive models that may have influenced the final results, which we excluded. Notably, due to the missing GG genotype of the population in some studies, ORs and 95% CIs could not be calculated, which may make the final combined ORs and 95% CIs less plausible.

It is worth pointing out that we cannot disregard the effect of sample size on the final results. The updated meta-analysis is more prone to type I errors.^[75]^ When the number of subjects in a given study is small, and the sample size of patients is small, random errors may lead to erroneous results; conversely, if the number of patients is large, the results usually converge to the true value.^[76]^ Some of the positive results may stem from random errors and are not actual effects of genes. In particular, the minor gene (G) is more susceptible when the gene frequencies are low in the population. Wetterslev et al^[33]^ first introduced the concept of TSA and applied it in an updated meta-analysis, which then began to be widely applied.^[34,77,78]^ Consequently, we examine the effect of random error on the results by TSA. The sample size was estimated to overcome the deficiencies of the study and minimize false-positive results. In the sample size assessment of all tumor types (Fig. 5A), the *Z* curve transcends the TSA cutoff curve, but the cumulative *Z* value does not exceed the required information size. In the sample size assessment of GC (Fig. 5B), the *Z* curve transcends the TSA cutoff curve, and the cumulative *Z* value exceeds the required information size. These results demonstrated that the sample size of this study is sufficient, and the results are accurate without more experimental confirmation. We conducted in-silico analysis at the end of our study to validate our conclusions. As the results revealed, the expression level of TLR4 in GC tissues was higher than in normal tissues, but the difference was not significant, which does not refute our conclusion because other polymorphisms affect the expression of TLR4. If some polymorphisms of TLR4 are under-expressed in GC tissues, it may lead to insignificant differences in the presentation of the whole gene in different tissues; these polymorphisms may be relevant to reduced GC risk, as confirmed by some studies.^[79,80]^

Of course, we should also be conscious of the constraints of this study. Firstly, only Caucasians and Asians were involved in this study. Other ethnic groups were under-researched, resulting in an incomplete analysis of ethnic differences in the association between rs4986790 and tumor risk. Secondly, of the five models we studied, the recessive and homozygous models had some of the studied GG genotypes missing in the population (the genotype frequency of GG was 0 in both the case and control groups), reducing the number of studies included in both models that could be used for data analysis. The reason may be the low gene frequency of the minor allele G in the natural population (MAF: G = 0.0579). Finally, the presence of non-neoplastic diseases in a few study controls may impact the final results, specifically when these diseases correlate with the TLR4 polymorphism rs4986790.

A broader range of races could be included in future studies while controlling for risk factors such as age or gender. Since potential biases and confounders cannot be wholly excluded throughout the study, we need larger, better-designed, and more comprehensive studies in the future. Research on TLR4 has been relatively advanced, including the association of TLR4 with cancer and the molecular mechanism of the TLR4 gene causing cancer. However, there are fewer studies on clinical efficacy, and further research is needed to determine whether the TLR4 gene can be a breakthrough in cancer treatment in the future. At the same time, we cannot overlook the role of TLR4 in tumor proliferation. Its mechanism of action in the inflammatory process of tumor development needs further to reveal, which can better serve the future of cancer prevention and therapy.

## 5. Conclusion

There was a meaningful correlation between rs4986790 (A > G) and tumors. Among them, rs4986790 minor allele G may increase the risk of GC development. Further subgroup analysis revealed that this association exists in Caucasians and Asians.

## Acknowledgments

Thank you to Jiangxi Provincial Health Commission for the financial support.

## Author contributions

**Conceptualization:** Guomin Zhu.

**Data curation:** Qiang Xiao.

**Formal analysis:** Qiang Xiao, Jian Chen, Hu Cai.

**Funding acquisition:** Guomin Zhu.

**Investigation:** Jian Chen, Hu Cai.

**Methodology:** Qiang Xiao.

**Project administration:** Guomin Zhu.

**Resources:** Jian Chen, ShuKun Zeng.

**Software:** Qiang Xiao.

**Supervision:** ShuKun Zeng.

**Validation:** Jian Chen, ShuKun Zeng.

**Writing – original draft:** Qiang Xiao.

**Writing – review & editing:** Jian Chen.

## Supplementary Material


