# Supplementary material for: An updated systematic review of the association between the TLR4 polymorphism rs4986790 and cancers risk
Source: Medicine (Baltimore). 2022 Oct 21;101(42):e31247. doi: 10.1097/MD.0000000000031247 (PMC9592503; doi:10.1097/MD.0000000000031247)
Supplement: Supplementary file 1 [file medi-101-e31247-s001.pdf]

PubMed:(n=23)

(((((Case-Control Study[Title/Abstract]) OR (Case-Control Studies[Title/Abstract])) OR (Studies, Case-Control[Title/Abstract])) OR (Study, Case-Control[Title/Abstract])) AND ((((((((Single Nucleotide Polymorphism[MeSH Terms])) OR (Nucleotide Polymorphism, Single[Title/Abstract])) OR (Nucleotide Polymorphisms, Single[Title/Abstract])) OR (Polymorphisms, Single Nucleotide[Title/Abstract]) OR (Single Nucleotide Polymorphisms[Title/Abstract])) OR (SNPs[Title/Abstract])) OR (Polymorphism, Single Nucleotide[Title/Abstract])))) AND ((((((((((((Neoplasia[MeSH Terms])) OR (Tumor[Title/Abstract])) OR (Neoplasm[Title/Abstract])) OR (Tumors[Title/Abstract])) OR (Neoplasias[Title/Abstract])) OR (Cancer[Title/Abstract])) OR (Malignant Neoplasm[Title/Abstract])) OR (Malignancy[Title/Abstract])) OR (Malignancies[Title/Abstract])) OR (Malignant Neoplasms[Title/Abstract])) OR (Neoplasm, Malignant[Title/Abstract])) OR (Neoplasms, Malignant[Title/Abstract])) AND (((((rs4986790[Title/Abstract]) OR (TLR4 Receptor[Title/Abstract])) OR (Toll Like Receptor 4[Title/Abstract]))

Web of science(n=47)

((((((TI=(Neoplas*)) OR TI=(Cancer*)) OR TI=(Tumor*)) OR TI=(Malignanc*)) OR TI=(“Malignant Neoplasm*”)) OR TI=(Carcinoma)) AND (((TI=(rs4986790)) OR TI=(TLR4)) OR TI=(Toll Like Receptor 4)) AND (((TS=(“Single Nucleotide Polymorphism*”)) OR TS=(snp)) OR TS=(Polymorphism*)) AND (((TS=(“Case-Control Stud*”)) OR TS=(“Stud*, Case-Control”)) OR TS=(“Case-Comparison Stud*”))

Embase(N:65)

('malignant neoplasm'/exp OR 'cancer':ti,ab,kw OR 'cancers':ti,ab,kw OR 'malignant neoplasia':ti,ab,kw OR 'malignant neoplasm':ti,ab,kw OR 'malignant neoplastic disease':ti,ab,kw OR 'malignant tumor':ti,ab,kw OR 'malignant tumour':ti,ab,kw OR 'neoplasia, malignant':ti,ab,kw OR 'tumor, malignant':ti,ab,kw OR 'tumour, malignant':ti,ab,kw) AND ('single nucleotide polymorphism'/exp OR 'polymorphism, single nucleotide':ti,ab,kw OR 'single nucleotide polymorphism':ti,ab,kw OR 'single nucleotide variant':ti,ab,kw OR 'single nucleotide variation':ti,ab,kw) AND (rs4986790:ti,ab OR 'toll like receptor 4'/exp OR 'cd284 antigen':ti,ab,kw OR 'tlr 4':ti,ab,kw OR 'tlr4':ti,ab,kw OR 'antigen cd284':ti,ab,kw OR 'toll like receptor 4':ti,ab,kw OR 'toll-like receptor 4':ti,ab,kw) AND ('case control study'/exp OR 'case control study':ti,ab,kw OR 'case-control studies':ti,ab,kw OR 'case-control study':ti,ab,kw OR 'control study, case':ti,ab,kw)

CNKI (n=3)

（（主题：肿瘤）OR（主题：癌症）OR（主题：恶性肿瘤）OR（主题：癌））and （（主题：单核苷酸）OR（主题：多态性）OR （主题：单核苷酸多肽现象））and （主题：TLR4） and （主题：病例对照研究）

Wan Fang(n=42)

主题:(TLR4) and 主题:(多态性) and 主题:(病例对照研究) and 主题:(癌)
