# Supplementary material for: An updated systematic review of the association between the TLR4 polymorphism rs4986790 and cancers risk
Source: Medicine (Baltimore). 2022 Oct 21;101(42):e31247. doi: 10.1097/MD.0000000000031247 (PMC9592503; doi:10.1097/MD.0000000000031247)
Supplement: Supplementary file 2 [file medi-101-e31247-s002.pdf]

Supplemental Table 1. Aggregate assessment outcomes of study quality.

author	year	Selection				Comparability	Exposure			Score
RS4986790		Item 1	Item 2	Item 3	Item 4	Item 5	Item 6	Item 7	Item 8	
Zheng	2004	*	*	*	*	**		*		7
Hellmig	2005	*	*	*	*	*		*		6
Chen	2005	*	*	*	*	**	*	*		8
Boraska	2006	*	*	*	*	**		*		7
Landi	2006	*	*		*	*	*	*		6
Forrest	2006	*	*	*	*	**		*		7
Nieters	2006	*	*	*	*	**		*		7
Garza-Gonzalez	2007	*	*			**	*	*		6
Hold (A-group)	2007	*	*	*	*	**		*		7
(B-group)	2007	*	*	*	*	**		*		7
(C-group)	2007	*	*	*	*	**		*		7
Cheng	2007	*	*		*	**		*		6
Santini	2008	*	*	*	*	**		*		7
Ture-Ozdemir	2008	*	*			**	*	*		6
Trejo-de la	2008	*	*		*	**		*		6
Wang	2009	*	*	*	*	**		*		7
Etokebe	2009	*	*		*	*	*	*		6
Pandey	2009	*	*		*	**		*		6
Purdue	2009	*	*	*	*	**		*		7
Balistreri	2010	*	*	*	*	**		*		7
Ashton	2010	*	*	*	*	**		*		7
Gast	2011	*	*	*	*	*		*		6
Davoodi	2011	*	*		*	**		*		6
shui	2012	*	*	*	*	**		*		7
de Oliveira	2012	*	*		*	**		*		6
de Oliveira	2013	*	*		*	**		*		6
Qadri	2013	*	*	*		**		*		6
shen	2013	*	*		*	**		*		6
Pimentel-Nunes	2013	*	*		*	**		*		6
Companioni	2014	*	*	*	*	**	*	*		8
Kutikhin	2014	*	*	*	*	**		*		7
Omrane	2014	*	*		*	**		*		6
Kurt	2016	*	*		*	**		*		6
Li	2017	*	*		*	**		*		6
Pandy	2019	*	*		*	**		*		6
Aref	2020	*	*		*	**	*	*		7
Eed	2020	*	*		*	**		*		6
Neamatallah	2020	*	*	*	*	**		*		7
Quirino	2021	*	*	*	*	*		*		6
Reilly	2021	*	*			**	*	*		6
Banescu	2022	*	*	*	*	**		*		7

Item 1, Adequate case definition; **Item 2**, Representativeness of the cases; **Item 3**, Selection of controls; **Item 4**, Determination of controls; **Item 5**, Comparability of cases and controls on the design or analysis; **Item 6**, Ascertainment of exposure; **Item 7**, Same method of ascertainment for cases and controls; **Item 8**, Non-response rate; one star (*) indicates 1 point.
