# Supplementary material for: An updated systematic review of the association between the TLR4 polymorphism rs4986790 and cancers risk
Source: Medicine (Baltimore). 2022 Oct 21;101(42):e31247. doi: 10.1097/MD.0000000000031247 (PMC9592503; doi:10.1097/MD.0000000000031247)
Supplement: Supplementary file 3 [file medi-101-e31247-s003.pdf]

Supplemental Table 2. Results of quantitative analysis of publication bias in the included literature.

Model	Number of studies	Begg's test		Egger's test	
		Z value	P	T value	P
Dominant	38	0. 57	0. 580	1. 70	0. 097
Recessive	22	0. 06	0. 955	−0. 11	0. 910
Homozygous	22	0. 28	0. 778	−0. 07	0. 947
Heterozygous	38	0. 43	0. 669	1. 59	0. 119
Additive	38	0. 28	0. 782	1. 60	0. 117
