# Supplementary material for: An updated systematic review of the association between the TLR4 polymorphism rs4986790 and cancers risk
Source: Medicine (Baltimore). 2022 Oct 21;101(42):e31247. doi: 10.1097/MD.0000000000031247 (PMC9592503; doi:10.1097/MD.0000000000031247)
Supplement: Supplementary file 4 [file medi-101-e31247-s004.pdf]

Supplemental Table 3. Preliminary appraisal of sources of heterogeneity using meta-regression.

Covariates	Number of dummy variables	dominant model	recessive model	homozygous model	heterozygous model	additive model
RS4986790						
Ethnicity	3	0.951	0.264	0.294	0.767	0.704
Source of controls	3	0.184	0.221	0.204	0.298	0.129
Cancer	5	0.810	0.646	0.567	0.729	0.925
Genotyping methods	8	0.047	0.075	0.067	0.093	0.029
Cons		0.456	0.473	0.516	0.618	0.337
